# A Statistical Approach toward Biofilms Hydrophilicity Characterization: Integrating ANOVA, DOE, and PCA for Starch/Glycerol/CMC Films Case Study

**DOI:** 10.1021/acsabm.5c00510

**Published:** 2025-05-07

**Authors:** Lisa Rita Magnaghi, Esther Trigueros, Raffaela Biesuz

**Affiliations:** # Università degli Studi di Pavia, Dipartimento di Chimica, Viale Taramelli 12, Pavia 27100, Italy; § INSTM, Unità di Ricerca di Pavia, Via G. Giusti 9, Firenze 50121, Italy

**Keywords:** Hydrophilicity, Moisture, Solubility, Bioplastics, Chemometrics, Design of Experiments, Principal Components Analysis, Analysis of Variance

## Abstract

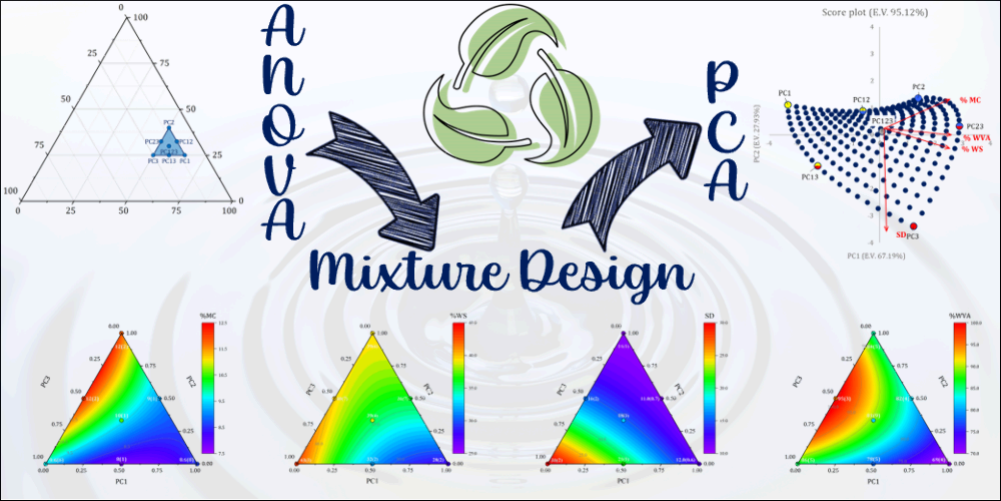

A combined statistical approach significantly improves the reliability of gravimetric assessments of hydrophilicity in starch/glycerol/carboxymethylcellulose biofilms. This study reveals that traditional methods for evaluating hydrophilicity-related properties are limited by inconsistent experimental conditions and inadequate statistical design. By systematically varying film composition within a pseudocomponent domain and applying ANOVA, Mixture Design, and Principal Components Analysis, we demonstrate that composition-related effects far exceed intrinsic method variability. The results underscore the influence of components on hydrophilic behavior, offering robust and predictive models across the experimental domain. This findings-oriented approach provides a rational framework to optimize formulations, ensuring reproducible and meaningful assessments of the hydrophilicity.

Gravimetric methods for hydrophilicity-related properties assessment, namely, Moisture Content (MC), Water Solubility (WS), Swelling Degree (SD), and Water Vapor Absorption (WVA), nowadays represent mandatory procedures in bioplastics characterization. Even during a quick survey of recent literature (2021–2025), anyone can verify that at least two of these procedures are always performed when proposing a novel biofilm, especially if starch is selected as the main component and casting deposition is used.^1−13^ Such a widespread diffusion of these methods is strongly related to the low resistance of starch-based materials toward water or humidity. Any novel starch-based material does need to exhibit a significant improvement in this feature compared with traditional materials.

Despite the fundamental role and the popularity of these procedures in the recent literature, a critical evaluation of the methodologies immediately reveals several weaknesses that may strongly limit the reliability of the results. Just to give an immediate hint, most of the descriptions start with the sentence “the parameter was measured according to the method proposed by Reference with slight modifications”.^2,4−6,8−10,12,14−16^ In fact, the general method requires subsequently drying and water or water vapor exposure steps and weight assessment, but within this workflow, almost every procedure differs in the experimental conditions. The main differences can be found in specimen dimensions and weight, drying conditions (*T* and duration), and exposure conditions (*T*, duration, stirring, solid–water ratio). As a consequence, only results presented in the same paper can be reliably compared, while no comparison is allowed between materials proposed by different research groups.

Besides modifications in experimental conditions, most of the papers are poorly designed from a statistical point of view, thus posing serious doubts on result reliability. MC, WS, SD, and WVA are always reported as an average value together with standard deviation, but how these values are computed is often unclear. The number of replicates is only sometimes mentioned,^9,10,12,13,15,17,18^ while generally omitted;^2,4,5,7,8,14,16^ the type of replicates is always omitted, i.e., whether the raw data comes from independently prepared samples or different regions of the same sample, and the reported digits often overcome the significant ones. It goes without saying that both casting deposition and gravimetric methods suffer from intrinsically high variability due to several uncontrolled irreproducibility sources. Nevertheless, a proper application of univariate (Analysis of Variance, ANOVA) and multivariate (Design of Experiments, DOE)^19^ data treatments do allow us to consider the intrinsic variability and to highlight significant effects.

A rigorous approach is seldomly followed in the literature: ANOVA coupled with Duncan or Turkey’s test is generally applied to demonstrate the significant effect of material composition modification, such as the use of tailored additives on which the paper is focused,^4,5,7,9,10,12,13,15−18^ while DOE has been applied a few times to improve operational parameters in material synthesis.^2,6,8^ In this context, only two papers propose the use of Mixture or Mixture and Process Design to jointly improve bioplastics composition and operational parameters, but none of them focus on the optimization of films’ hydrophilicity.^20,21^

Taking into consideration the faster and faster technological improvements and the widespread diffusion of both more sophisticated instruments for material characterization and for data analysis or optimization, we raise the issue of the dated and poorly designed methodologies in use for hydrophilicity-related property assessment, and we propose a rational approach to improve the reliability of these characterizations by merging univariate and multivariate data analysis. In this Letter, starch/glycerol/carboxymethylcellulose (CMC) biofilms at variable composition are presented as a case study and MC, WS, SD, and WVA are assessed according to one of the methods proposed in the literature. Variability both within prepared biofilms, possibly related to preparation irreproducibility, and among biofilms of different compositions is assessed by ANOVA and, when significant differences can be attributed to composition changes, Mixture Design is used to model each hydrophilicity-related property separately. After a single models’ development and validation, the overall hydrophilic behavior of starch/gly/CMC biofilms at variable composition is summarized by Principal Components Analysis (PCA).^22^

As far as experimental condition variability, resulting in incomparable results, there are actually no correct conditions, but we did our best to properly report all the experimental details in order to make the assessments reproducible by anyone. Further investigations on the effect of experimental conditions on outcomes would be beneficial to improve the literature background.

Experimentally, starch/gly/CMC biofilms are synthesized by casting deposition using rice starch and Carbocel MA200 as CMC (Lamberti Spa, Gallarate - 21013 VA, Italy) and applying the method previously optimized by Magnaghi and coauthors.^21^ Glycerol is dispersed in 20 mL of H_2_O under heating and stirring; starch and CMC powders are then added once the solution reaches 85 °C. The specific amounts for each component are defined according to the Mixture Design approach, as described in the previous section. The aqueous mixture is kept under heating (85 °C) and stirring (600 rpm) for 20 min, covering the beaker with a watch glass to reduce solvent evaporation. After homogenization, the mixture is poured into food-grade silicone molds (diameter = 5.5 cm) and allowed to cool down for 1 h at RT; next, air is removed by placing a 2 × 2 mold in a dryer connected to a vacuum pump and keeping the samples under vacuum for 1 h. Finally, the 2 × 2 mold is transferred in a laboratory oven, previously heated at 65 °C, dried for 20 h, and then cooled at RT (22 °C) for at least 24 h before peeling from the mold.

Native moisture content (MC) is computed as the weight differences between specimens equilibrated at room temperature and humidity (*w*_initial_) and after drying for 24 h at 75 °C (*w*_dry_), as reported in the following equation.
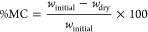


Water solubility (WS) is computed as the weight differences between dried specimens (24 h at 75 °C) before (*w*_dry_) and after (*w*_dry2_) 24 h of immersion in 20 mL of H_2_O at 20 °C under gently soaking, as reported in the following equation.
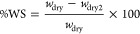


Swelling degree (SD) is computed as the weight differences between dried specimens (24 h at 75 °C, *w*_dry_) and swollen ones after 24 h of immersion in 20 mL of H_2_O at 20 °C under gently soaking (*w*_swelling_), corrected for weight loss during water soaking due to partial solubility of the film (*w*_solubility_), as reported in the following equation. Excess water is removed by sorbent paper after water soaking and before weight measurement.
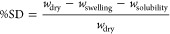


Water vapor adsorption (WVA) is computed as the weight differences between dried specimens (24 h at 75 °C) before (*w*_dry_) and after (*w*_vapor_) a 4-day exposure in a closed atmosphere at 97% of relative humidity (obtained using saturated K_2_SO_4_ solution), as reported in the following equation.
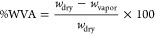


For all the characterizations, the samples’ reproducibility for each composition is checked by comparing the results obtained on different sections of different samples by ANOVA; later, the significance of composition-related effects is demonstrated by comparing average results on the different sections for each independently prepared sample per composition.

Once the sample homogeneity (ANOVA between different sections), composition reproducibility (ANOVA between replicates), and significance of the composition-related effects are verified, the so-acquired MC, WS, SD, and WVA data are used to compute the coefficients for the polynomial models, one for each hydrophilicity-related property (*y*). Since the inclusion of independent replicates improves model robustness and reliability, the average value between sections per sample prepared per composition under investigation is submitted to Multiple Linear Regression (MLR) to be used in the coefficients’ computation. All chemometrics data analyses are performed using CAT software.^23^

The four models are validated by comparing experimental and predicted values at the center of the experimental domain, taking into account confidence intervals to be associated with both the values. Response surfaces in the entire mixture domain are computed to evaluate the composition-related effects on the responses under investigation.

Lastly, a comprehensive overview of hydrophilic behavior of starch/gly/CMC biofilms at variable compositions is provided by PCA: the computed models are used to predict the four response values in each point of the mixture domain (setting 0.05 as minimum variation for the component amount); the so-obtained data set, i.e., predicted MC, WS, SD, and WVA for the entire domain under investigation, is submitted to PCA after autoscaling to visualize correlations between different hydrophilicity-related properties.

Hydrophilicity-related properties are investigated for starch/gly/CMC biofilms within a well-defined range of compositions, represented in Figure 1: starch content is varied between 65% and 50%, glycerol is between 40% and 25%, and CMC is between 25% and 10%. Different methodologies allow the application of Mixture Design when a fraction of the mixture domain is investigated: in this case, we opted to define the so-called pseudocomponents (PCs) as extreme compositions exhibiting the higher content of each component and, thus, the pseudocomponents domain, highlighted in blue in Figure 1.^21^ These boundaries are defined starting from a literature composition (60% starch, 30% glycerol, 10% CMC) and performing preliminary experiments to evaluate how much this composition could be varied while still obtaining suitable films, thus defining the explorable section of the experimental domain.^21^

**Figure 1:**
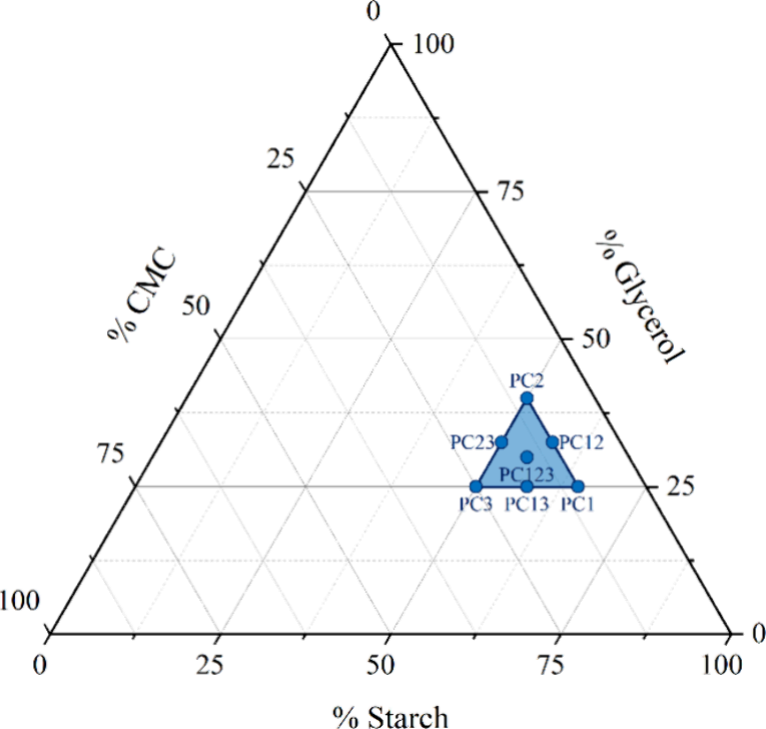
Pseudocomponents domain definition within the entire starch/gly/CMC mixture domain.

Following this approach, the mixture is investigated in the pseudocomponents domain that retains all the geometrical features of the original domain and thus allows its description by Simplex Centroid Design (SCD).^24^ It goes without saying that, after validation, the computed models can be considered reliable only within the pseudocomponent domain, while they cannot be extrapolated to the rest of the original mixture domain.

Therefore, the seven compositions reported in Table 1 are synthesized: the first 6 compositions are used as a training set to build the models according to the general equation reported below, while the central point serves to validate the developed models.
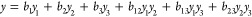


**Table 1:** Training (1–6) and Test (7) Composition Definitions According to the Simplex Centroid Design Applied in the Pseudocomponents Domain

PCs	% Starch	% Glycerol	% CMC
PC1	65	25	10
PC2	50	40	10
PC3	50	25	25
PC12	57.5	32.5	10
PC13	57.5	25	17.5
PC23	50	32.5	17.5
PC123	54.45	29.7	14.85

At least 12 replicates are synthesized for each pseudocomponent and cut into four sections to jointly evaluate films by ANOVA reproducibility and composition-related effects for each hydrophilicity-related property, as summarized in Figure 2. In all cases, the preparation and gravimetric methods can be considered reproducible since the variability within sections of the same sample does not significantly differ from variability within sections coming from different samples, as described in Figure S1. Conversely, sample composition influences the four properties under investigation, resulting in variations significantly above the intrinsic variability of the preparation and gravimetric methods, as summarized in Figure S2.

**Figure 2:**
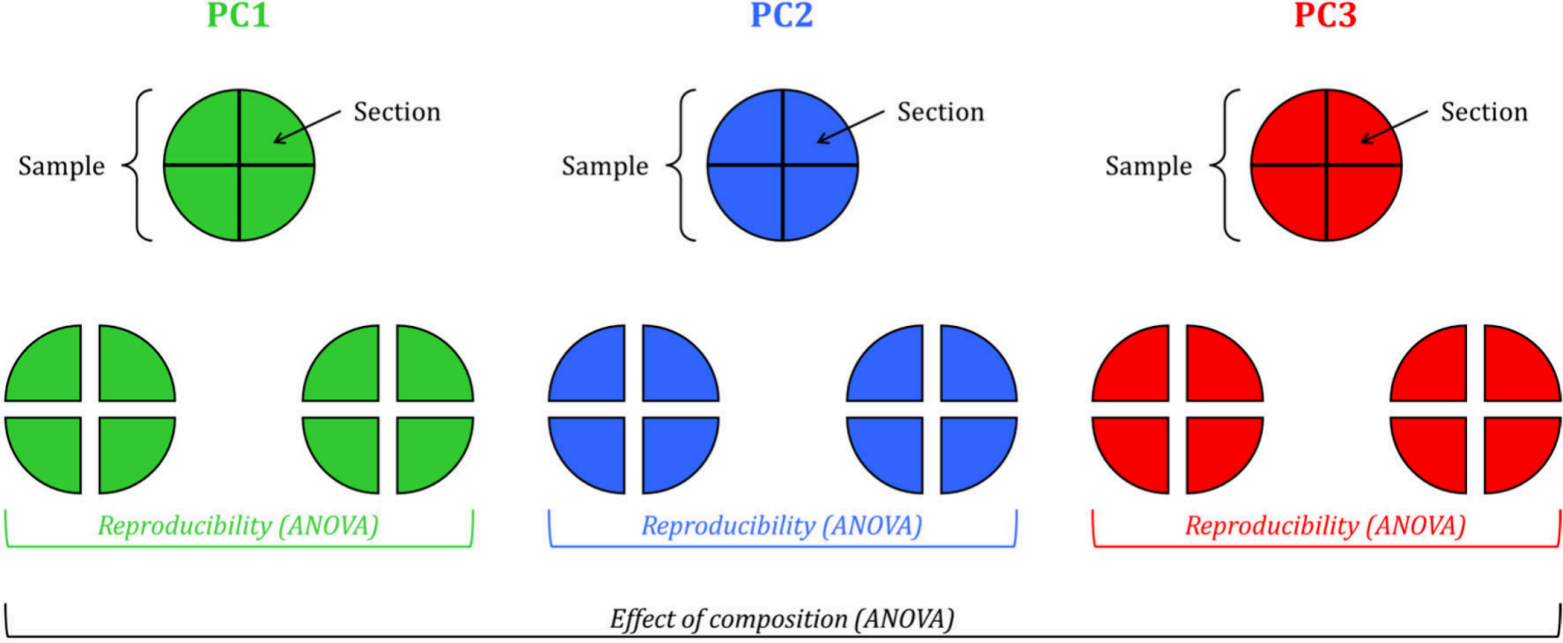
Data analysis workflow for reproducibility and composition-related effects assessment by ANOVA.

Table 2 reports the average values for Moisture Content (MC), Water Solubility (WS), Swelling Degree (SD), and Water Vapor Absorption (WVA) for each composition in Table 1, computed by using four sections of three independently synthesized samples (*n* = 12) for each.

**Table 2:** Average Values of MC, WS, SD, and WVA for Each Composition in Table 1 (*n* = 12), Including Standard Deviation in Brackets

PCs	MC (%)	WS (%)	SD	WVA (%)
PC1	8.6(8)	28(2)	12.0(6)	69(4)
PC2	12(2)	39(6)	11(1)	85(5)
PC3	9.6(6)	43(3)	31(2)	87(2)
PC12	9(1)	36(7)	11.0(7)	82(4)
PC13	8(2)	32(2)	21(1)	78(5)
PC23	12(2)	40(7)	16(2)	95(3)
PC123	11(1)	39(4)	18(3)	81(9)

The experimental values of MC, WS, SD, and WVA are employed to compute the coefficients (Figure S3) for the models dedicated to each property by Multiple Linear Regression (MLR). The models are then validated by comparing the experimental and predicted values, considering the confidence interval at 95% confidence level for both values, for each property at the center of the pseudocomponents domain, i.e., for PC123, as shown in Table S1. Once validated, the models are used to predict the trend of each property in the pseudocomponent domain and to build the response surfaces in Figure 3.

**Figure 3:**
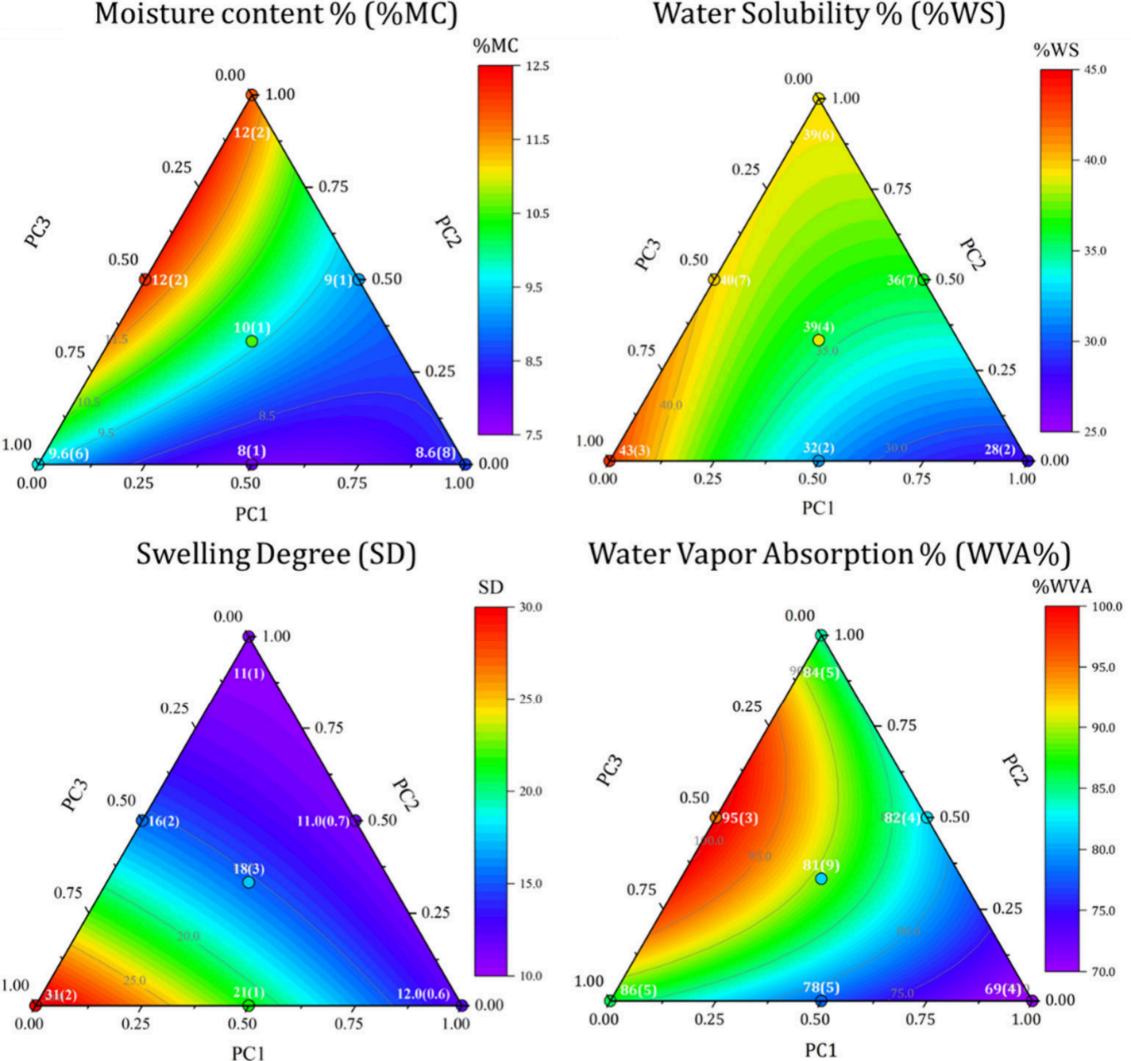
Computed response surfaces for Moisture Content (%MC), Water Solubility (%WS), Swelling Degree (SD), and Water Vapor Adsorption (%WVA) within the pseudocomponents domain and comparison with the experimental values for the compositions in Table 1.

We can easily observe that both MC and WVA increase for decreasing starch content, thus moving from PC1 composition to the opposite side of the pseudocomponents domain. The starch-dependent behavior is more evident for the first property, in which a stiffer decrease is observed, rather than for the latter one, in which the changes are more gradual. The opposite trend can be noticed for WS that is a minimum in biofilms containing high percentages of starch and strongly decreases with the increase of both glycerol and CMC content. Lastly, SD shows a peculiar behavior almost completely related to the CMC content, with a 3-fold increase in PC3 samples rather than PC1 or PC2.

Since the separate evaluation of the four response surfaces might be laborious, we tried to summarize the hydrophilic behavior of the films by submitting the predicted values for the four responses in the entire domain to PCA. Autoscaling is applied to normalize different responses in terms of mean value and standard deviation. The loading plot in Figure 4 clearly depicts the correlation among the different properties investigated: WS and WVA are strongly directly correlated to one another and also, less evidently, to MC while SD exhibits a completely different and uncorrelated behavior. As far as composition is concerned, samples with maximum starch content show the least values of MC, WVA, and WS, while the higher values are registered for PC23, namely, the samples containing high amounts of both glycerol and CMC. Oppositely, the highest SD is shown by CMC-rich samples and the lowest values are registered for all the samples including low amounts of CMC, regardless of starch and glycerol content. These findings can be easily justified considering the chemical properties of the raw materials: starch is poorly soluble in water and tends to exhibit hydrophobic behavior, and both CMC and glycerol are water-soluble, with the first exhibiting swelling properties and the latter being extremely hydrophilic.

**Figure 4:**
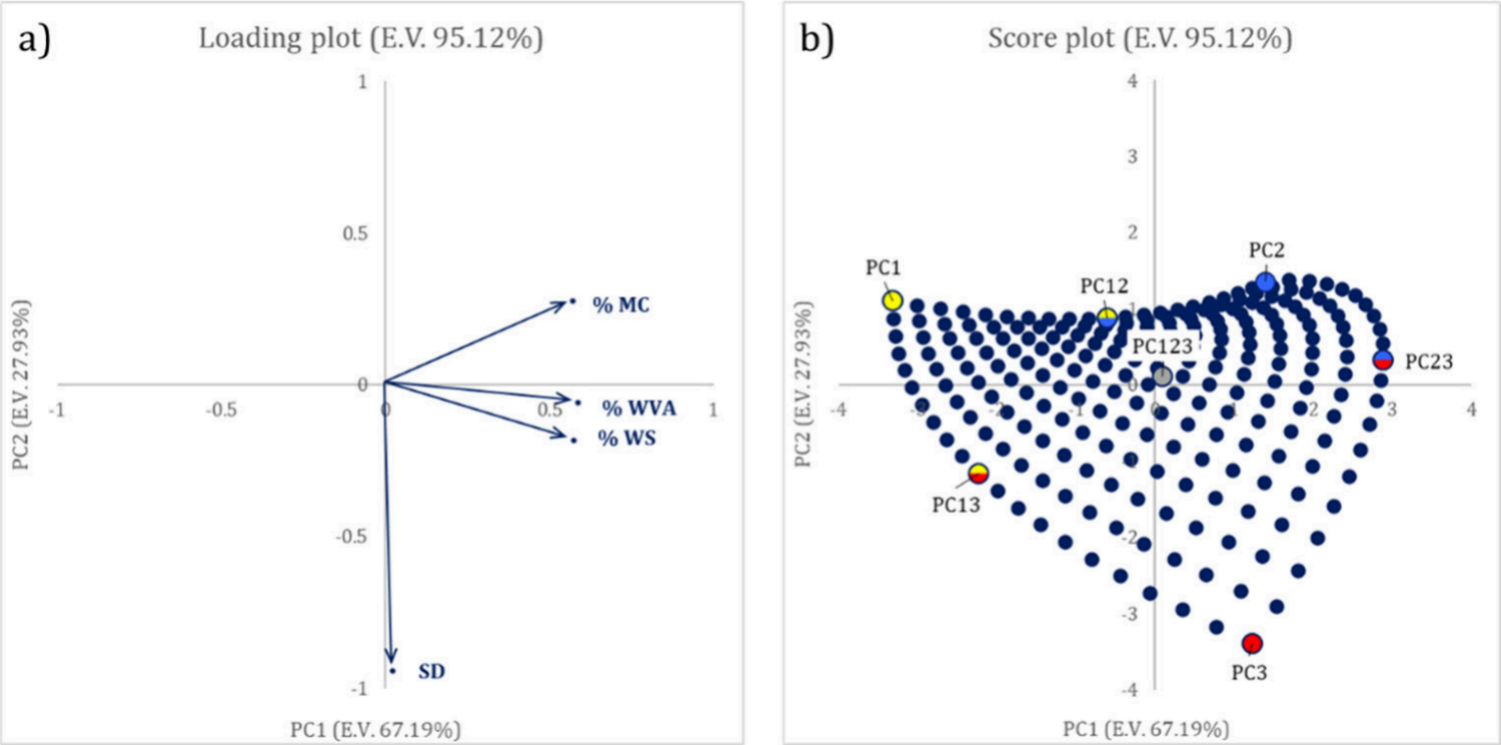
Loading (a) and score (b) plots for the first two components of the PCA model on predicted MC, WS, SD, and WVA values of the compositions within the pseudocomponents domain.

In conclusion, we are presenting a statistical approach that merges univariate and multivariate data analysis to improve the scientific reliability of gravimetric methods for hydrophilicity-related properties, to cope with the intrinsic variability of these procedures, nevertheless recognizing significant effects related to biofilm composition, in this case study, or other parameters, and finally to model the trend of these properties in the experimental domain of interest and to jointly visualize the hydrophilic behavior of the samples at a glance.

The case study proposed includes three main components in the biofilms, but the same approach can also be extended to materials including more compounds. Additionally, the PCA application to compare different properties can be easily extended to a much higher number of features without significant computational effort. For this reason, we believe that this approach can be easily transferred to more complex compositions or characterizations.

## Supporting Information Available

Figure S1: Comparison between MC, WS, SD, and WVA registered for sections of the same or of different samples for each composition and ANOVA results; Figure S2: Comparison between MC, WS, SD, and WVA registered for sections of samples with different compositions and ANOVA results; Figure S3: Computed coefficients for the model referred to MC, WS, SD, and WVA; Table S1: Comparison between experimental and fitted MC, WS, SD, and WVA values for PC123 samples, considering the respective confidence intervals (PDF)

## Abbreviations

MC: moisture content
WS: water solubility
SD: swelling degree
WVA: water vapor absorption
ANOVA: analysis of variance
DOE: design of experiments
CMC: carboxymethylcellulose
PCA: principal components analysis
PC: pseudocomponent
SCD: simplex centroid design
MLR: multiple linear regression
