# Supplementary material for: A Statistical Approach toward Biofilms Hydrophilicity Characterization: Integrating ANOVA, DOE, and PCA for Starch/Glycerol/CMC Films Case Study
Source: ACS Appl Bio Mater. 2025 May 7;8(5):3691–6. doi: 10.1021/acsabm.5c00510 (PMC12093360; doi:10.1021/acsabm.5c00510)
Supplement: Supplementary file 1 — mt5c00510_si_001.pdf [file mt5c00510_si_001.pdf]

Supporting Information

A Statistical Approach towards Biofilms Hydrophilicity Characterization: Integrating ANOVA, DOE, and PCA for Starch/Glycerol/CMC Films Case Study

Lisa Rita Magnaghi*[#,§], Esther Trigueros[#], Raffaela Biesuz[#,§]

[#]Università degli Studi di Pavia, Dipartimento di Chimica, Viale Taramelli 12, Pavia, 27100, Italy

[§]INSTM, Unità di Ricerca di Pavia, Via G. Giusti 9, Firenze, 50121, Italy

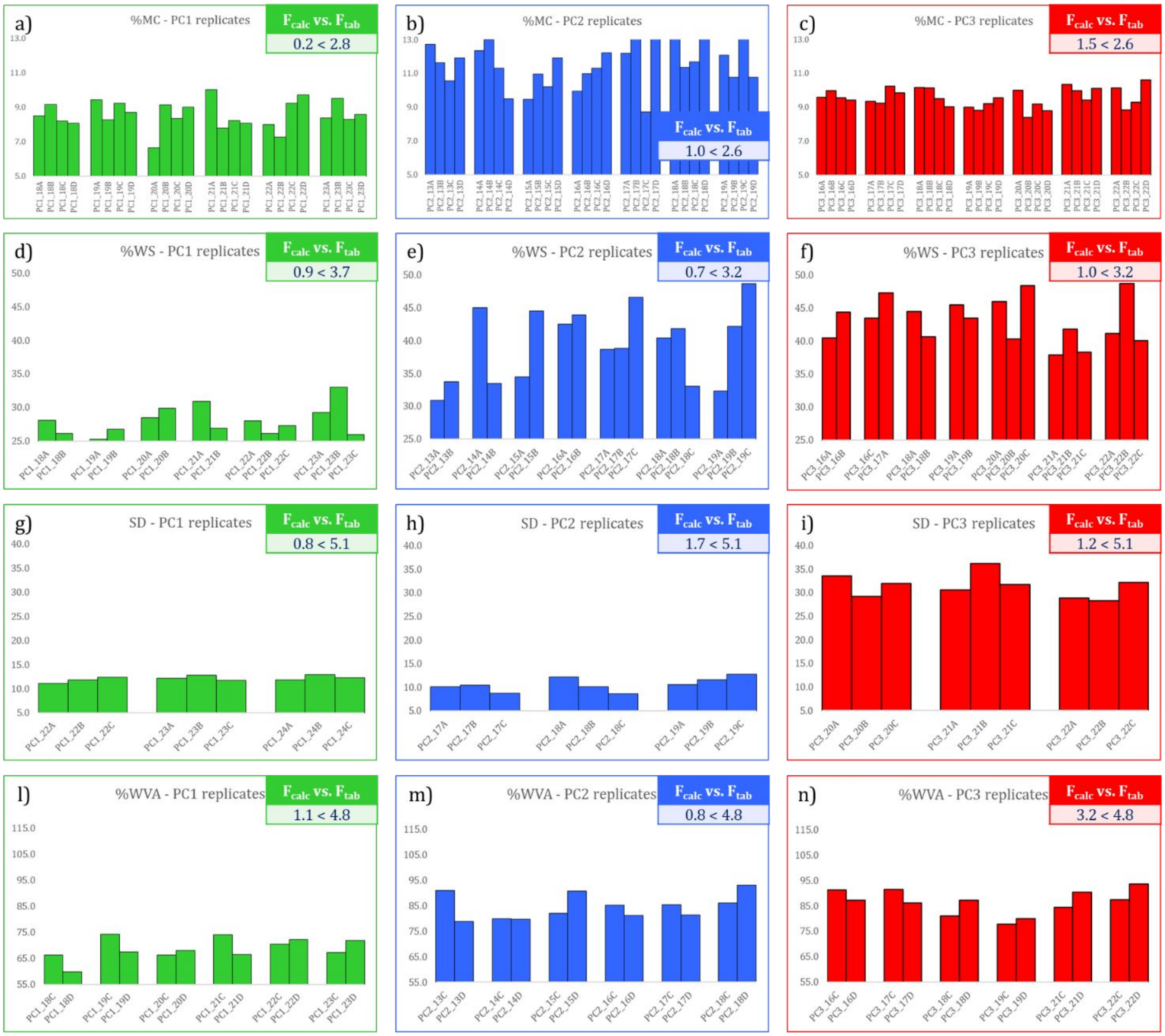

FIGURE S1: Comparison between MC, WS, SD and WVA registered for sections of the same or of different samples per each composition and ANOVA results

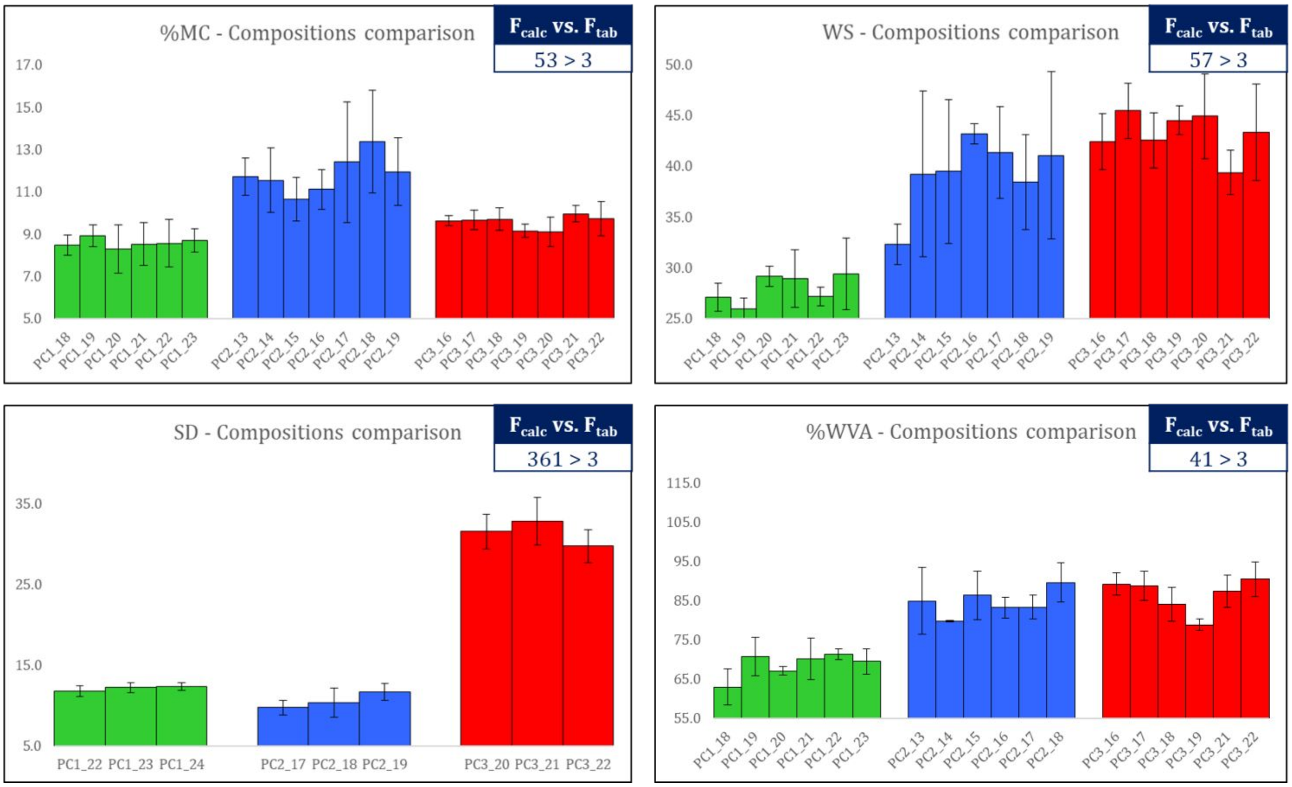

FIGURE S2: Comparison between MC, WS, SD and WVA registered for sections of samples with different compositions and ANOVA results

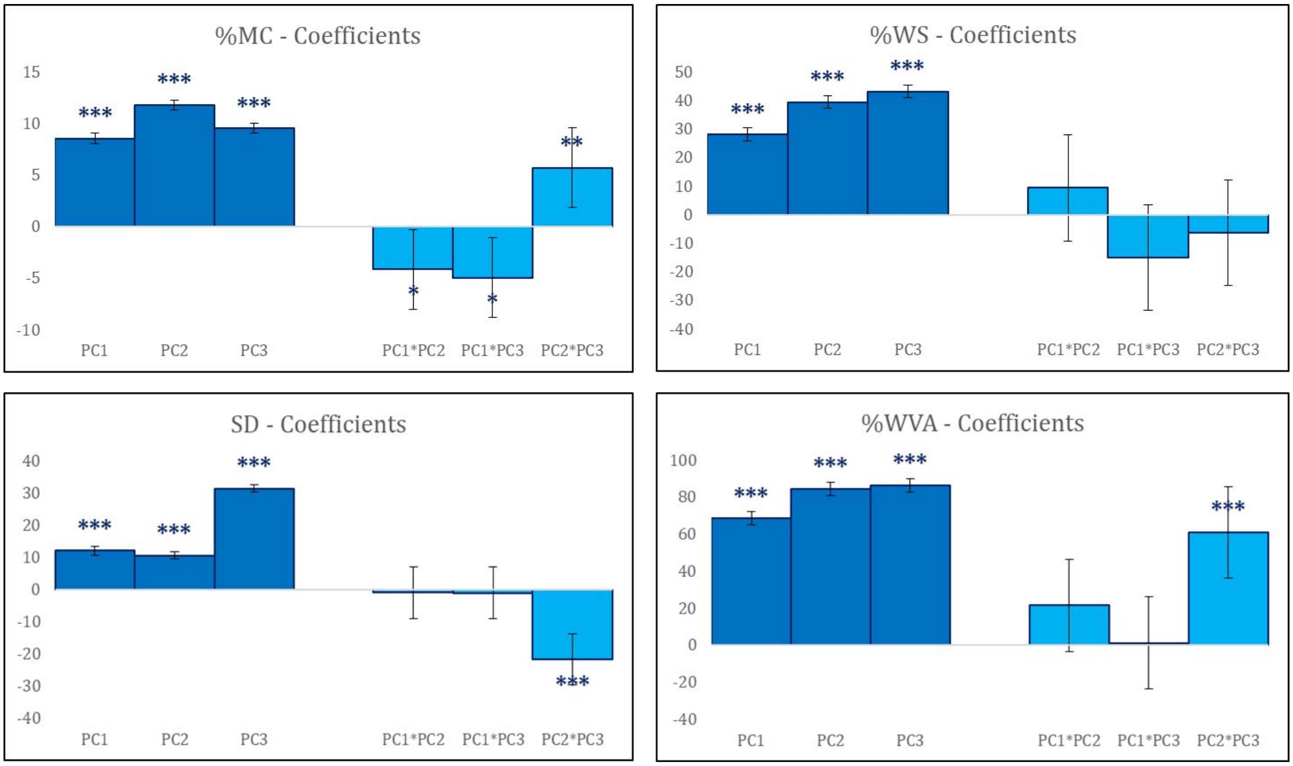

FIGURE S3: Computed coefficients for the model referred to MC, WS, SD and WVA

TABLE S1: Comparison between experimental and fitted MC, WS, SD and WVA values for PC123 samples, considering the respective confidence intervals (CI)

Property	Value	Lower limit CI	Mean value	Upper limit CI
%MC	Experimental	10.1	10.5	11.0
	Predicted	8.8	9.5	10.2
%WS	Experimental	36	39	41
	Predicted	32	35	38
SD	Experimental	15	18	20
	Predicted	14	15	17
%WVA	Experimental	75	81	88
	Predicted	84	88	92
